# Prognostic Relevance of Type 4a Myocardial Infarction and Periprocedural Myocardial Injury in Patients With Non–ST-Segment–Elevation Myocardial Infarction

**DOI:** 10.1161/CIRCULATIONAHA.124.070729

**Published:** 2025-02-19

**Authors:** Matteo Armillotta, Luca Bergamaschi, Pasquale Paolisso, Marta Belmonte, Francesco Angeli, Angelo Sansonetti, Andrea Stefanizzi, Davide Bertolini, Francesca Bodega, Sara Amicone, Lisa Canton, Damiano Fedele, Nicole Suma, Andrea Impellizzeri, Francesco Pio Tattilo, Daniele Cavallo, Ornella Di Iuorio, Khrystyna Ryabenko, Andrea Rinaldi, Gabriele Ghetti, Francesco Saia, Cinzia Marrozzini, Gianni Casella, Paola Rucci, Alberto Foà, Carmine Pizzi

**Affiliations:** 1 Department of Medical and Surgical Sciences, Alma Mater Studiorum, University of Bologna, Italy (M.A., L.B., F.A., A. Sansonetti, D.B., F.B., S.A., L.C., D.F., N.S., A.I., F.P.T., D.C., O.D.I., K.R., F.S., A.F., C.P.).; 2 Cardiovascular Division, Morgagni–Pierantoni University Hospital, Forlì, Italy (M.A., L.B., F.A., S.A., L.C., D.F., C.P.).; 3 Cardiology Unit, Sant’Andrea University Hospital, Rome, Italy (P.P.).; 4 Department of Advanced Biomedical Sciences, University Federico II, Naples, Italy (M.B.).; 5 Cardiovascular Center Aalst, OLV Hospital, Aalst, Belgium (M.B.).; 6 Cardiology Unit, IRCCS Azienda Ospedaliera-Universitaria di Bologna, Italy (A. Sansonetti, D.B., F.B., N.S., A.I., F.P.T., D.C., O.D.I., K.R., A.R., G.G., F.S., C.M., A.F.).; 7 Cardiology Division, Parma University Hospital, Azienda Ospedaliero-Universitaria di Parma, Italy (A. Stefanizzi).; 8 Unit of Cardiology, Maggiore Hospital, Bologna, Italy (G.C.).; 9 Division of Hygiene and Biostatistics, Department of Biomedical and Neuromotor Sciences, Alma Mater Studiorum, University of Bologna, Italy (P.R.).

**Keywords:** myocardial infarction, non-ST elevated myocardial infarction, percutaneous coronary intervention, treatment outcomes, troponin

## Abstract

**BACKGROUND:**

Periprocedural myocardial injury (PMI) with or without type 4a myocardial infarction (MI) might occur in patients with non–ST-segment–elevation MI (NSTEMI) after percutaneous coronary intervention (PCI). This study investigated the incidence and prognostic relevance of these events, according to current definitions, in patients with NSTEMI undergoing PCI. The best cardiac troponin I (cTnI) threshold of PMI for prognostic stratification is also suggested.

**METHODS:**

Consecutive patients with NSTEMI from January 2017 to April 2022 undergoing PCI with stable or falling pre-PCI cTnI levels were enrolled. According to the Fourth Universal Definition of Myocardial Infarction, the study population was stratified into those experiencing (1) PMI with type 4a MI, (2) PMI without type 4a MI, or (3) no PMI. Post-PCI cTnI increase >20% with an absolute postprocedural value of ≥5 times the 99th percentile upper reference limit within 48 hours after PCI was used to define PMI. The primary end point was 1-year all-cause mortality, and the secondary end point consisted of major adverse cardiovascular events at 1 year, including all-cause mortality, nonfatal reinfarction, urgent revascularization, nonfatal ischemic stroke, and hospitalization for heart failure. Internal validation was performed in patients enrolled between May 2022 and April 2023.

**RESULTS:**

Among 1412 patients with NSTEMI undergoing PCI with stable or falling cTnI levels at baseline, 240 (17%) experienced PMI with type 4a MI, 288 (20.4%) experienced PMI without type 4a MI, and 884 (62.6%) experienced no PMI. PMI was associated with an increased risk of adverse clinical outcomes, with patients with type 4a MI demonstrating the highest rates of 1-year all-cause mortality and major adverse cardiovascular events. A post-PCI ΔcTnI >20% but ≤40% showed similar outcomes to patients without PMI, whereas >40% was identified as the optimal threshold for prognostically relevant PMI, confirmed in an internal validation cohort of 305 patients.

**CONCLUSIONS:**

Periprocedural ischemic events were frequent in patients with NSTEMI undergoing PCI with prognostic implications. A post-PCI ΔcTnI >40%, combined with an absolute postprocedural value of ≥5 times the 99th percentile upper reference limit, was identified as the optimal threshold for diagnosing prognostically relevant PMI. Recognizing these events may improve risk stratification and management of patients with NSTEMI.

**Clinical Perspective**

**What Is New?**

- This study is the first to investigate the incidence and prognostic impact of periprocedural myocardial injury (PMI) with and without type 4a myocardial infarction after non–ST-segment–elevation myocardial infarction (NSTEMI) in patients undergoing percutaneous coronary intervention (PCI).
- A considerable number of patients with NSTEMI developed PMI with or without type 4a myocardial infarction after PCI. These events were associated with a significantly increased risk of 1-year adverse outcomes and occurred more frequently than in patients with chronic coronary syndromes.
- A post-PCI change in troponin I >40%, along with an absolute postprocedural value of ≥5 times the 99th percentile upper reference limit, was identified as the optimal threshold for defining prognostically relevant PMI.

**What Are the Clinical Implications?**

- These findings suggest that PMI with or without type 4a myocardial infarction significantly affects outcomes in patients with NSTEMI undergoing PCI.
- The newly identified post-PCI troponin I change threshold >40% for diagnosing a prognostically relevant PMI might aid risk stratification and management of patients with NSTEMI.
- The suggested criterion for defining prognostically relevant PMI, a >40% increase within 3 to 6 hours post-PCI combined with an absolute postprocedural value exceeding 5 times the 99th percentile upper reference limit, could serve as a valuable clinical end point in future research on the management and outcomes of patients with NSTEMI.

Non–ST-segment–elevation myocardial infarction (NSTEMI) is a common cardiac condition that contributes to a substantial portion of hospital admissions and cardiovascular morbidity and mortality worldwide.^1,2^ Although NSTEMI incidence has been increasing over the years,^1,3^ percutaneous coronary intervention (PCI) has significantly improved prognosis, reducing cardiovascular death and nonfatal myocardial infarction (MI), particularly in high-risk patients.^4–7^ The availability of new techniques and advances in medical therapy have lowered the occurrence of thrombotic complications during and after PCI, resulting in improved procedural success rates and patient outcomes.^8^ Nonetheless, elevation of cardiac troponin (cTn) after PCI is still frequently observed, especially with the use of high-sensitivity troponin tests.^9,10^

The definition and prognostic role of periprocedural myocardial injury (PMI) and type 4a MI are well established in patients with chronic coronary syndromes undergoing PCI with nonelevated baseline cTn levels.^9–11^ Conversely, the incidence, interpretation, and prognostic relevance of PMI and type 4a MI, as currently defined by the Fourth Universal Definition of Myocardial Infarction (UDMI), in patients with NSTEMI (thus with elevated baseline troponin) are still unknown, as stated in the recent consensus document of the European Society of Cardiology Working Group on Cellular Biology of the Heart and the European Association of Percutaneous Cardiovascular Interventions.^9^ Moreover, the post-PCI ΔcTn cutoff of 20% used to define type 4a MI and PMI after NSTEMI is based on consensus expert opinion, not supported by robust evidence.^12^ A standardized definition of periprocedural ischemic events based on scientific evidence, with prognostic relevance and applicable in clinical practice, is highly warranted.^9^

This study aims to investigate (1) the incidence of PMI with and without type 4a MI in patients with NSTEMI undergoing PCI; (2) the prognostic relevance of these periprocedural ischemic events defined according to current consensus (post-PCI ΔcTn >20% with an absolute postprocedural value of ≥5 times the 99th percentile upper reference limit [URL])^12^; and (3) the best post-PCI cTn change threshold for prognostic stratification of patients with PMI.

## Methods

### Study Design and Population

The present study is a prespecified subanalysis of the observational prospective registry AMIPE (Acute Myocardial Infarction, Prognostic and Therapeutic Evaluation; NCT03883711), evaluating the outcomes of patients admitted with acute MI to S. Orsola-Malpighi and Maggiore Hospitals of the Bologna metropolitan area. In the present analysis, we included consecutive patients with NSTEMI undergoing PCI between January 1, 2017, and April 30, 2022.

The diagnosis of NSTEMI was based on the Fourth UDMI, and patients were managed according to current guidelines.^1,12,13^ From hospital admission, all patients underwent serial measurements of cTn I (cTnI). As required for the diagnosis of periprocedural MI or myocardial injury, patients were eligible if pre-PCI cTnI levels were stable (variation ≤20%) or falling.^12^ Exclusion criteria for the present study were unavailability of serial cTnI measurements, detection of rising pre-PCI cTnI levels (unstable, variation >20%), incomplete data at the 1-year follow-up, and lack of informed consent.

All the results were internally validated in a cohort of consecutive patients with NSTEMI undergoing PCI enrolled at the same 2 centers between May 1, 2022, and April 30, 2023.

The protocol was approved by the institutional review board (registration No. 600/2018/Oss/AOUBo). The present study was conducted according to the Declaration of Helsinki; all patients were informed about their participation in the study and provided informed consent for the anonymous publication of scientific data. Details of data collection are provided in the Supplemental Methods. The datasets used or analyzed during the current study are available from the corresponding author on reasonable request.

### Measurement of cTn levels

From January 1, 2017, to September 4, 2018, conventional cTnI levels were measured by chemiluminescent immunoassay for antigen detection (Beckman Coulter Access AccuTnI+3 assay), with a 99th percentile URL of 40 ng/L for both men and women. From September 5, 2018, onward, high-sensitivity cTnI (hs-cTnI) was collected with the Access hsTnI assay (Beckman Coulter), which has a URL of 19.8 ng/L for men and 11.6 ng/L for women.

Before PCI, cTnI measurements were performed at hospital admission (0 hours), every 3 hours until the cTnI peak was reached, and within 1 hour before coronary angiography (baseline cTnI). After PCI, ≥3 measurements of cTnI were obtained: at the end of PCI and after 3 and 6 hours. In case of an increase in post-PCI cTnI or if clinically indicated (eg, new ischemic symptoms or electrocardiographic changes), repeated measurements were obtained every 3 hours to assess peak post-PCI levels within the first 48 hours after PCI.

Preprocedural and postprocedural cTnI levels were used for the current analysis. Post-PCI ΔcTnI (Δ%) was calculated as [(post-PCI peak cTnI – baseline cTnI)/baseline cTnI]×100.

### Definitions of PMI and Type 4a MI

Type 4a MI and PMI were adjudicated by 2 independent experts (P.P. and A.S.) using all clinical and instrumental information collected during the index hospitalization. According to the Fourth UDMI, in patients with elevated baseline cTnI who had stable (variation ≤20%) or falling cTnI levels, the post-PCI cTnI increase >20% with an absolute postprocedural value of ≥5 times the 99th percentile URL was used for the definition of PMI, taking into account the differences between conventional and high-sensitivity troponin assays, as well as sex-specific differences for hs-cTnI.^12^ Type 4a MI was diagnosed in the presence of PMI plus 1 of the following elements: (1) new ischemic ECG changes, (2) development of new pathological Q waves, (3) imaging evidence of new loss of viable myocardium or new regional wall motion abnormality in a pattern consistent with an ischemic origin, or (4) angiographic findings consistent with a procedural flow-limiting complication such as coronary dissection, loss of a side branch, slow flow, thrombus, or distal embolization.^12^

Per study protocol, all patients of the final study population underwent a standard 12-lead ECG at the time of first medical contact, on arrival at the cardiac intensive care unit, before PCI, after PCI within 1 hour (on return to the cardiac intensive care unit), and every morning until discharge. All patients also underwent at least one 2-dimensional transthoracic echocardiography at the time of NSTEMI diagnosis and at least one 2-dimensional transthoracic echocardiography within 48 hours after PCI. Furthermore, if clinically indicated, the patient underwent additional ECG and echocardiography. Further details were specified in the Supplemental Methods.

Based on the above definitions, patients were divided into 3 subgroups: (1) PMI with type 4a MI, (2) PMI without type 4a MI, and (3) no PMI.

### Follow-Up and End Points

Patients were followed up after discharge through outpatient visits or telephone contacts using a standard questionnaire.

The primary end point of the study was 1-year all-cause mortality. Cardiovascular deaths were defined as all deaths except those in which the exclusive primary underlying cause was noncardiovascular. The secondary end point was a composite of major adverse cardiovascular events (MACEs) at 1 year, including all-cause mortality, nonfatal reinfarction, urgent revascularization, nonfatal ischemic stroke, and hospitalization for heart failure. Patients were followed up until the first event for the calculation of MACE rates. The secondary end point definitions are reported in the Supplemental Methods.

### Statistical Analysis

Continuous variables were summarized as mean±SD or median and interquartile range according to the normality of the frequency distribution; categorical variables were summarized as absolute and percentage frequencies. The normality of the frequency distribution was assessed with the Shapiro-Wilk test.

Event-free survival was estimated with Kaplan-Meier curves and compared between groups with the log-rank test. Unadjusted and adjusted hazard ratios (HRs) for 1-year mortality and MACEs were calculated with Cox proportional hazard models. Age and peak pre-PCI cTnI levels, the most important known predictors of outcome in patients with NSTEMI, were used for adjustment. Age was considered a continuous variable, whereas peak pre-PCI cTnI concentration was log-transformed for normalization and expressed as a multiple of the URL. Other variables for adjustment (ie, confounders) were identified as the demographic and clinical characteristics associated with both outcome and exposure (the categorical variable no PMI/PMI without type 4a MI/PMI with type 4a MI). As potential confounders, we selected the main known predictors of outcome in patients with NSTEMI, specifically sex, diabetes, chronic obstructive pulmonary disease, complex PCI (the definition of complex PCI is provided in the Supplemental Methods), baseline creatinine, GRACE (Global Registry of Acute Coronary Events) score (coded as <140 or ≥140), left ventricular ejection fraction, complete revascularization (coded yes/no), prior MI, prior stroke, and peripheral artery disease. The identified confounding factors associated with both outcome and exposure were complete revascularization and complex PCI.

Receiver-operating characteristic curve analysis was performed to determine which measure—pre-PCI peak cTnI levels, post-PCI ΔcTnI, or post-PCI peak cTnI levels—was most accurate in predicting 1-year mortality for both assays, with the areas under the curves being compared using the DeLong test. The optimal cutoff for post-PCI ΔcTnI, balancing sensitivity and specificity, was identified using the maximum Youden index (sensitivity+specificity−1). In addition, a sensitivity analysis was conducted on patients undergoing hs-cTnI measurements and on those with an absolute postprocedural cTnI value ≥5 times the 99th percentile URL.

Univariable and multivariable logistic regression analyses were used to identify the baseline clinical and angiographic variables independently associated with PMI and type 4a MI. Variables with a significance level of 0.1 in univariable models were included in multivariable models.

All analyses were replicated in the internal validation cohort to test the robustness of the results obtained. Statistical analyses were performed with SPSS Statistics version 28.0.1.1 (IBM) and Stata version 17 (StataCorp). The significance level was set at *P*<0.05.

## Results

As shown in the study flowchart (Figure S1), 1581 patients admitted for NSTEMI undergoing PCI and included in the AMIPE registry were potentially eligible for the present study. Among those, 133 patients were excluded because of elevated and unstable cTnI levels (variation >20%) at the time of PCI; 25 patients because of unavailability of serial cTnI measurements; and 11 patients because of incomplete 1-year follow-up. The final sample consisted of 1412 patients with NSTEMI with stable or falling cTnI levels at baseline, all of whom had serial cTnI measurements, ECGs, and echocardiograms performed at the time points specified in the Methods section and complete 1-year follow-up data.

Baseline, angiographic, and procedural characteristics of patients with stable or falling pre-PCI cTnI levels compared with patients with unstable pre-PCI cTnI levels or no serial cTnI measurements are provided in Tables S1 and S2.

### Incidence of Periprocedural Ischemic Events According to Current Definitions

According to the Fourth UDMI, PMI occurred in 37.4% of patients (n=524), of whom 240 (17% of the overall cohort) met the criteria for type 4a MI. The remaining 62.6% of patients (n=884) did not experience any periprocedural ischemic events. Tables 1 and 2 show baseline clinical, angiographic, and procedural characteristics and discharge details of the 3 subgroups. Periprocedural ischemic events were assessed with hs-cTnI in most patients (n=755 patients, 53.5%); the remaining were evaluated with conventional cTnI (n=657 patients, 46.5%). The occurrence rate of PMI with and without type 4a MI did not differ between the periods of conventional cTnI testing and hs-cTnI testing (Table S3).

**Table 1.**
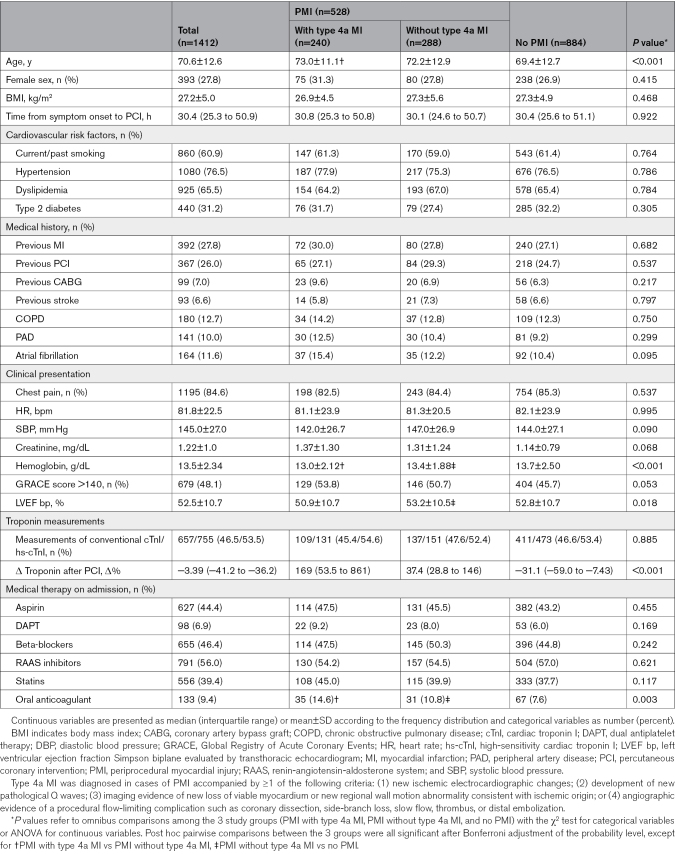
Baseline Characteristics of the Primary Cohort Study, Stratified According to Periprocedural Ischemic Events

**Table 2.**
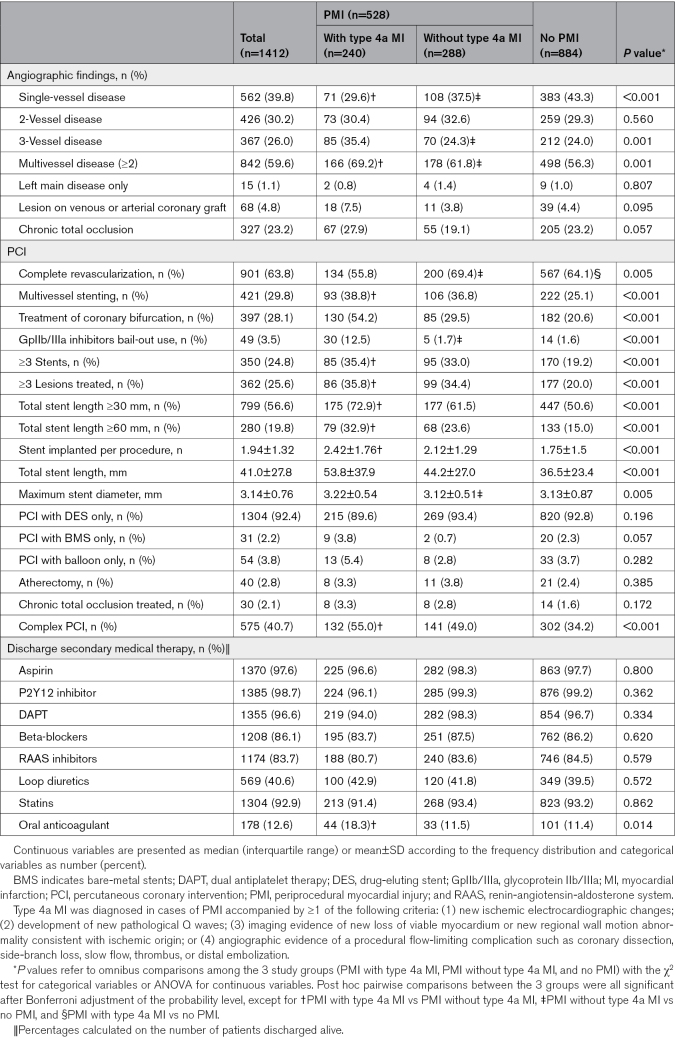
Description of the Angiographic Findings, Procedural Characteristics, and Discharge Details of the Primary Cohort Study, Stratified According to Periprocedural Ischemic Events

Among the 240 patients who developed PMI with type 4a MI: (1) 78.3% (n=188) exhibited new ischemic electrocardiographic changes or Q waves; (2) 27.9% (n=67) showed echocardiographic evidence of new loss of viable myocardium or new regional wall motion abnormality consistent with an ischemic origin, of whom 24 patients experienced a decrease in left ventricular ejection fraction ≥10% between the pre-PCI and post-PCI echocardiography; and (3) 79.6% (n=191) had angiographic findings indicating procedural flow-limiting complications. Fifty-two patients (21.7%) had evidence of periprocedural myocardial ischemia at ECG, echocardiography, and invasive coronary angiography. The angiographic findings associated with type 4a MI are presented in Table S4.

**Figure 1.**
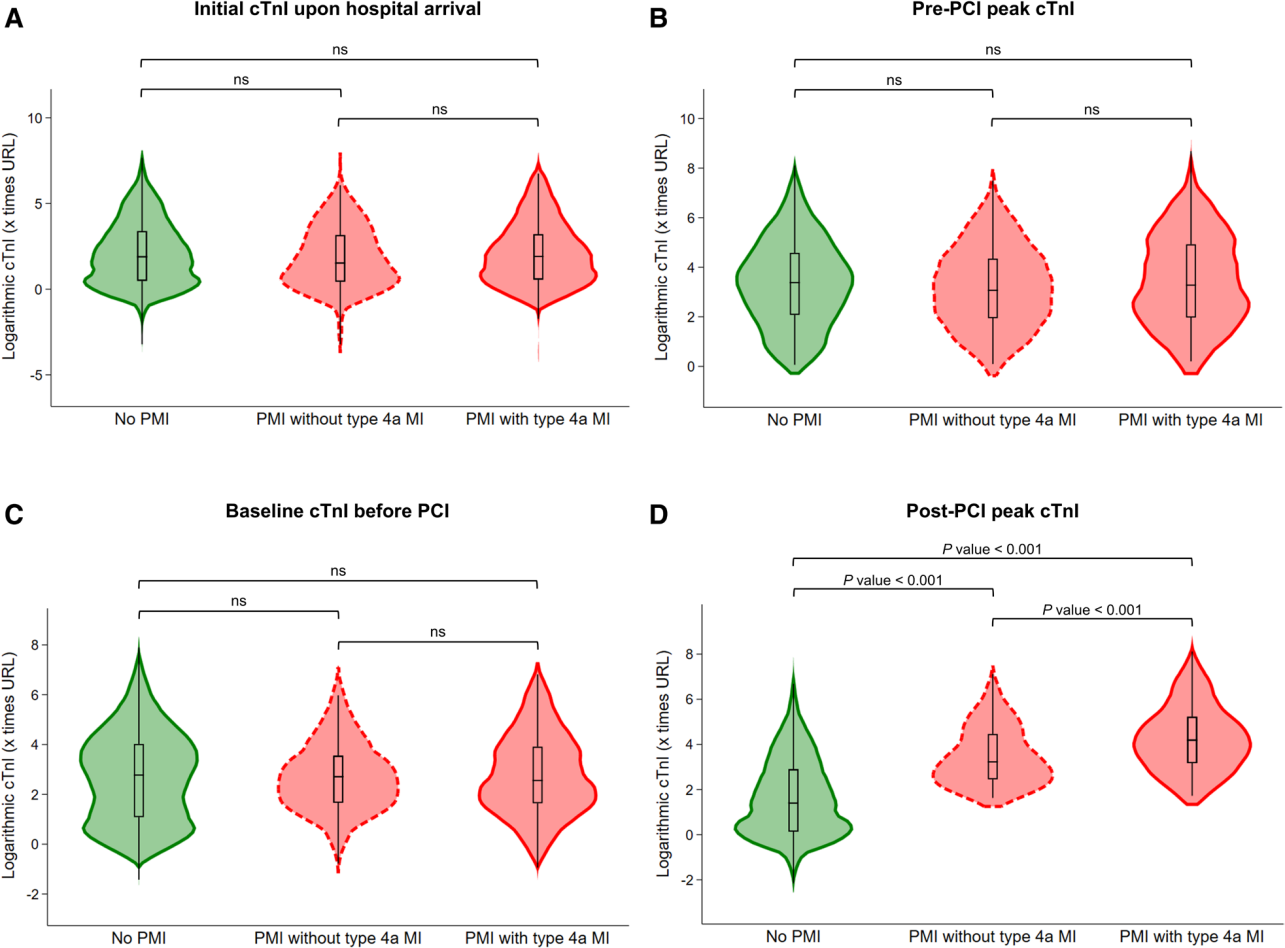
**Distributions of cTnI concentrations during hospitalization of the primary cohort study, stratified according to periprocedural ischemic events.** Violin plots of initial cardiac troponin I (cTnI) value on hospital arrival (**A**), peak cTnI value of the non–ST-segment–elevation myocardial infarction (MI) before percutaneous coronary intervention (PCI); **B**), baseline cTnI values within 1 hour before PCI (**C**), and peak cTnI value after PCI (**D**). cTnI concentrations were expressed as a multiple of the upper reference limit (URL) on a log scale, considering differences between conventional and high-sensitivity troponin I assays, as well as between sexes. Between-group differences were evaluated by Mann-Whitney *U* test. Sample sizes: overall cohort, n=1412; periprocedural myocardial injury (PMI) with type 4a MI, n=240; PMI without type 4a MI, n=288; and no PMI, n=884. Type 4a MI was diagnosed in cases of PMI accompanied by ≥1 of the following criteria: (1) new ischemic electrocardiographic changes; (2) development of new pathological Q waves; (3) imaging evidence of new loss of viable myocardium or new regional wall motion abnormality consistent with ischemic origin; or (4) angiographic evidence of a procedural flow-limiting complication such as coronary dissection, side-branch loss, slow flow, thrombus, or distal embolization. ns Indicates non-significant (*P*>0.05).

Distributions of cTnI concentrations during hospitalization, expressed as a multiple of the URL on a log scale, were presented by violin plots stratified according to the occurrence of periprocedural events (Figure 1), showing no significant differences in cTnI levels among the 3 subgroups at any time before PCI. Further details on pre-PCI and post-PCI cTnI values in the 3 subgroups are shown in Tables S5 and S6, stratified according to the specific type of cTnI measured.

### Prognostic Relevance of Periprocedural Ischemic Events According to Current Definitions

**Figure 2.**
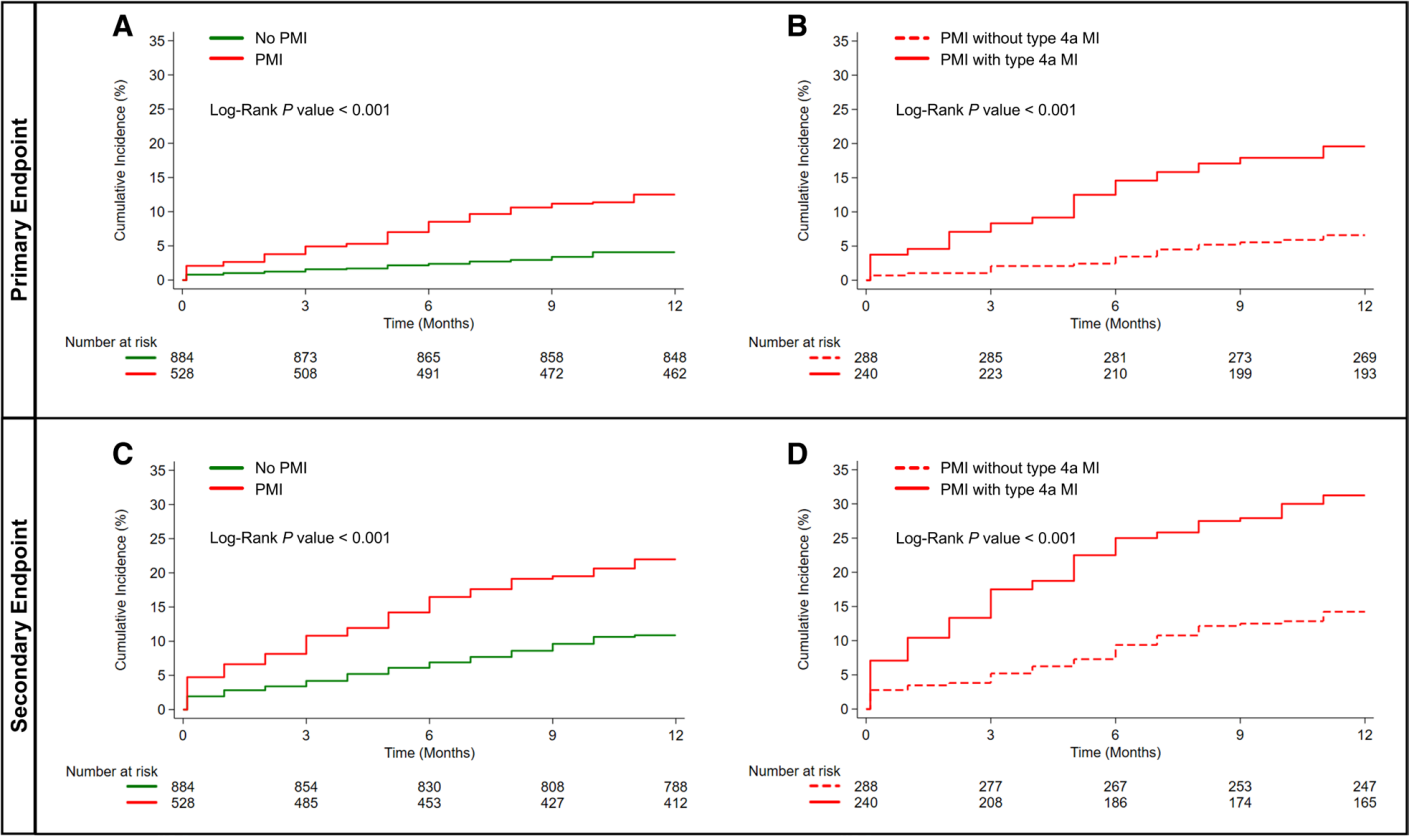
**Cumulative incidence curves for the primary and secondary end points at 1-year follow-up in the primary cohort study, stratified by periprocedural ischemic events. A**, Primary end point stratified by the presence of periprocedural myocardial injury (PMI). **B**, Further stratification of the primary end point in patients with PMI, comparing those with adjudicated type 4a myocardial infarction (MI) with those without type 4a MI. **C**, Secondary end point stratified by PMI. **D**, Further stratification of the secondary end point in patients with PMI, comparing those with adjudicated type 4a MI with those without type 4a MI. Type 4a MI was diagnosed in cases of PMI accompanied by ≥1 of the following criteria: (1) new ischemic electrocardiographic changes; (2) development of new pathological Q waves; (3) imaging evidence of new loss of viable myocardium or new regional wall motion abnormality consistent with ischemic origin; or (4) angiographic evidence of a procedural flow-limiting complication such as coronary dissection, side-branch loss, slow flow, thrombus, or distal embolization. The primary end point was 1-year all-cause mortality. The secondary end point was a composite of major adverse cardiovascular events at 1 year, including all-cause mortality, nonfatal reinfarction, urgent revascularization, nonfatal ischemic stroke, and hospitalization for heart failure.

The overall incidence of the primary and secondary end points was 7.2% and 15%, respectively. Patients who experienced PMI with type 4a MI had higher rates of all-cause mortality and MACEs at the 1-year follow-up compared with patients with PMI without type 4a MI criteria and those without periprocedural ischemic events (Table 3). Figure 2 shows Kaplan-Meier curves for the primary and secondary end points at the 1-year follow-up, illustrating worse outcomes for patients with PMI compared with those without PMI and for patients with adjudicated type 4a MI compared to those without adjudicated type 4a MI among patients with PMI.

**Table 3.**
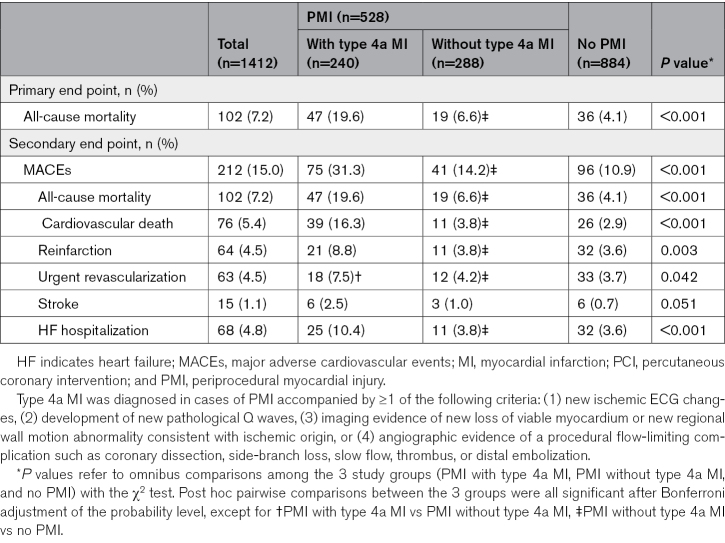
The 1-Year Outcomes After PCI of the Primary Cohort Study, Stratified According to Periprocedural Ischemic Events

In the multivariable Cox regression model, patients with PMI had a 3-fold increased risk of all-cause mortality (HR, 3.21 [95% CI, 2.14–4.82], *P*<0.001; adjusted HR [aHR], 2.68 [95% CI, 1.77–4.04], *P*<0.001) and an elevated risk of MACEs (HR, 1.54 [95% CI, 1.30–1.83], *P*<0.001; aHR, 1.39 [95% CI, 1.17–1.65], *P*<0.001) at the 1-year follow-up compared with those without periprocedural ischemic events. Furthermore, among patients with PMI, those meeting the criteria for type 4a MI had a 3-fold increased risk of all-cause mortality (HR, 3.23 [95% CI, 1.90–5.51], *P*<0.001; aHR, 2.94 [95% CI, 1.71–5.06], *P*<0.001) and a 2-fold increased risk of MACEs (HR, 2.48 [95% CI, 1.70–3.63], *P*<0.001; aHR, 2.25 [95% CI, 1.53–3.30], *P*<0.001) at the 1-year follow-up compared with those with PMI but without type 4a MI criteria (Table 4).

**Table 4.**
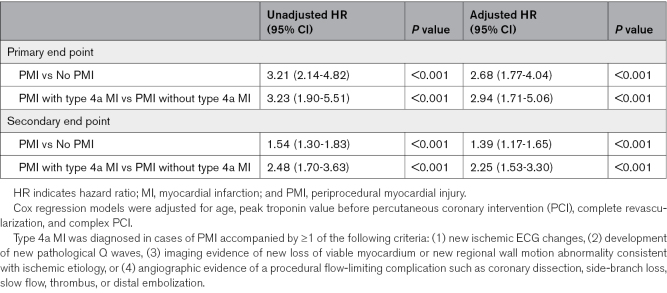
Cox Regression Model at 1-Year Follow-Up for Primary and Secondary End Points of the Primary Cohort Study, Stratified by Periprocedural Ischemic Events

### Defining the Optimal Prognostic Threshold for PMI

In the receiver-operating characteristic curve analysis, post-PCI ΔcTnI predicted the primary outcome more accurately than either pre-PCI or post-PCI peak cTnI levels (Figure S2). In the overall cohort, the optimal threshold for post-PCI ΔcTnI to predict 1-year all-cause mortality was >40%. This cutoff point provided an optimal balance between sensitivity (54.9%) and specificity (80.4%; Table S7). All patients were subsequently reclassified with these thresholds. The occurrence rates of post-PCI ΔcTnI levels of ≤20%, >20% but ≤40%, and >40% were similar between the periods of conventional cTnI testing and hs-cTnI testing (Table S8).

**Figure 3.**
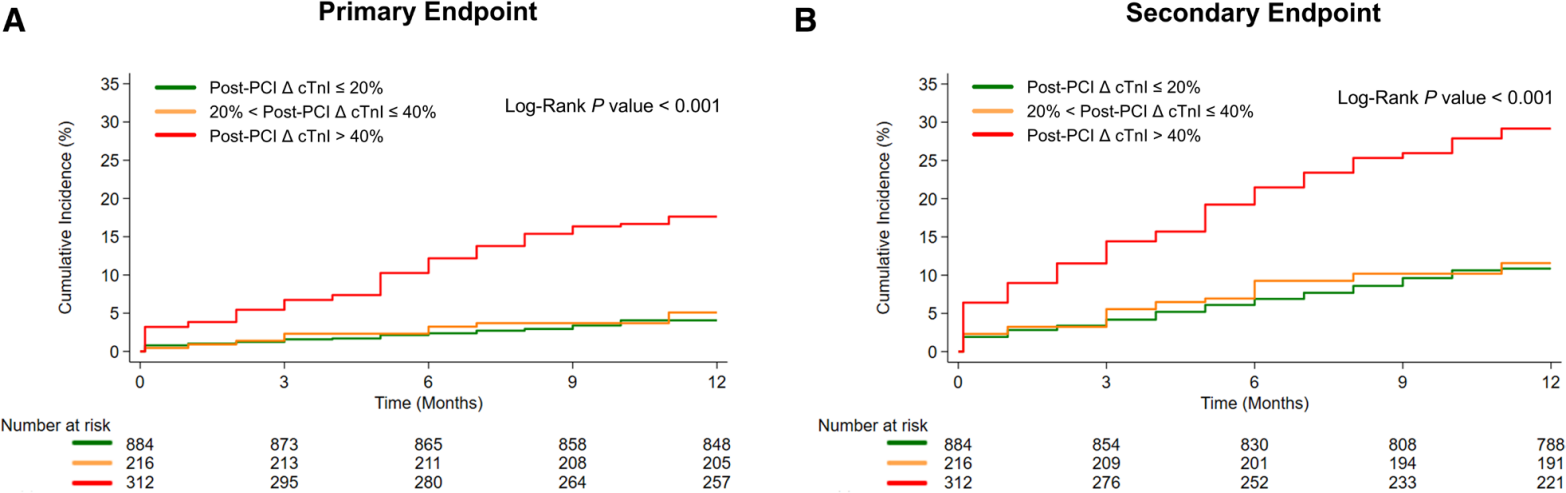
**Cumulative incidence curves for the primary and secondary end points at 1-year follow-up in the primary cohort study, stratified by post-PCI ΔcTnI levels of ≤20%, >20% but ≤40%, and >40%. A**, Primary end point stratified by post–percutaneous coronary intervention (PCI) change in cardiac troponin I (ΔcTnI) levels. **B**, Secondary end point stratified by post-PCI ΔcTnI levels. The primary end point was 1-year all-cause mortality. The secondary end point was a composite of major adverse cardiovascular events at 1 year, including all-cause mortality, non-fatal reinfarction, urgent revascularization, nonfatal ischemic stroke, and hospitalization for heart failure.

Among 528 patients in the PMI group defined according to Fourth UDMI criteria, 312 (22.1% of the overall population) had a post-PCI ΔcTnI >40%, and the remaining 216 patients had a post-PCI ΔcTnI >20% but ≤40%. Figure 3 illustrates the cumulative incidence curves for the primary and secondary end point at the 1-year follow-up, stratified by post-PCI ΔcTnI levels of ≤20% (no PMI subgroup), >20% but ≤40%, and >40%. The plots demonstrate worse outcomes for patients with a post-PCI ΔcTnI >40% for both end points.

Similarly, Cox regression analyses showed significantly higher risks for 1-year all-cause mortality (HR, 4.68 [95% CI, 3.07–7.12], *P*<0.001; aHR, 3.94 [95% CI, 2.57–6.04], *P*<0.001) and MACEs (HR, 3.02 [95% CI, 2.27–4.03], *P*<0.001; aHR, 2.79 [95% CI, 2.08–3.73], *P*<0.001) for patients with this newly defined PMI compared with those without periprocedural ischemic events (post-PCI ΔcTnI ≤20%). Conversely, patients with post-PCI ΔcTnI levels between 20% and 40% did not show an increased risk of 1-year all-cause mortality (HR, 1.25 [95% CI, 0.64–2.46], *P*=0.510; aHR, 1.04 [95% CI, 0.53–2.04], *P*=0.920) or MACEs (HR, 1.07 [95% CI, 0.69–1.67], *P*=0.748; aHR, 0.94 [95% CI, 0.60–1.46], *P*=0.782) compared with patients with post-PCI ΔcTnI levels ≤20% (Table 5).

**Table 5.**
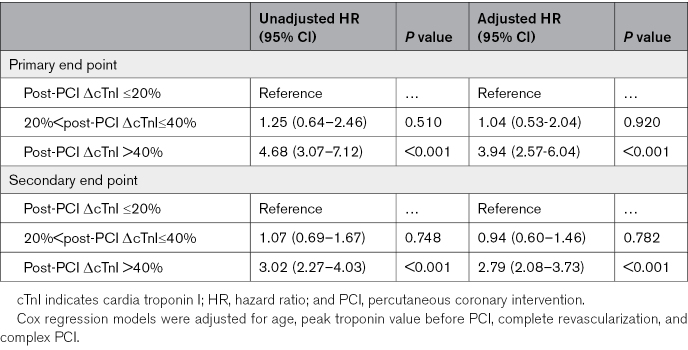
Cox Regression Model at 1-Year Follow-Up for Primary and Secondary End Points of the Primary Cohort Study, Stratified by Post-PCI ΔcTnI Levels of ≤20%, >20% but ≤40%, and >40%

These findings remained consistent in the sensitivity analysis on the subset of patients with an absolute postprocedural cTnI concentration ≥5 times the 99th percentile URL (81.2%, n=1146), as shown in Figure S3 and Table S9. Among these patients, the proportion with an adjudicated diagnosis of type 4a MI was 0% (0/622) in those with a post-PCI ΔcTnI ≤20%, 26.3% (56/213) in those with a post-PCI ΔcTnI >20% but ≤40%, and 59.2% (184/311) in those with a post-PCI ΔcTnI >40%.

### Internal Validation Cohort

The internal validation cohort consisted of 305 patients with NSTEMI undergoing PCI with stable or decreasing levels of hs-cTnI at the time of PCI. Baseline, angiographic, and procedural characteristics of this population are detailed in Tables S10 and S11.

The incidence of periprocedural ischemic complications was comparable to that observed in the primary cohort study, with PMI (defined according to the Fourth UDMI criteria) occurring in 37% of patients (n=113), of whom 48 (15.7% of the validation cohort) had adjudicated type 4a MI, whereas the remaining 63% (n=192) did not experience any periprocedural ischemic events. The overall incidence of primary and secondary end points was 7.5% and 15.1%, respectively (Table S12). Figure S4 shows the cumulative incidence curves for the primary and secondary end points at the 1-year follow-up in this patient cohort. Similar to the primary cohort study, patients with PMI had a higher risk of 1-year all-cause mortality and MACEs compared with those without in the validation cohort. In addition, among patients with PMI, those with adjudicated type 4a MI demonstrated an elevated risk for both outcomes compared with those with PMI but without type 4a MI criteria (Table S13).

After stratification of patients by post-PCI ΔcTnI levels of ≤20%, >20% but ≤40%, and >40%, only a post-PCI Δhs-cTnI >40% was independently associated with a higher risk of all-cause mortality (HR, 4.76 [95% CI, 1.97–11.48], *P*<0.001; aHR, 4.15 [95% CI, 1.67–10.30], *P*<0.001) and MACEs (HR, 3.30 [95% CI, 1.79–6.09], *P*<0.001; aHR, 2.78 [95% CI, 1.48–5.21], *P*=0.002) at the 1-year follow-up (Table S14; Figure S5).

Among the subgroup of patients with an absolute hs-cTnI concentration ≥5 times the URL (84.6%, n=258), the proportion of patients with an adjudicated diagnosis of type 4a MI was 0% (0/147) in those with a post-PCI ΔcTnI ≤20%, 4.9% (2/41) in those with a post-PCI ΔcTnI >20% but ≤40%, and 65.7% (46/70) in those with a post-PCI ΔcTnI >40%.

### Sensitivity Analysis in Patients Undergoing hs-cTnI Testing

In the sensitivity analysis of patients undergoing hs-cTnI measurements, PMI was associated with significantly higher risks of 1-year all-cause mortality and MACEs. Patients with PMI fulfilling the type 4a MI criteria had an even higher risk compared with those with PMI but without type 4a MI (Figure S6; Table S15). Receiver-operating characteristic curve analysis confirmed that a post-PCI Δhs-cTnI >40% was the optimal threshold for predicting 1-year all-cause mortality, demonstrating comparable sensitivity and specificity (Table S16). After stratification of patients by post-PCI Δhs-cTnI values, Cox regression models confirmed that only a post-PCI Δhs-cTnI >40% was significantly associated with worse outcomes (Figure S7; Table S17).

### Predictors of Periprocedural Ischemic Events After NSTEMI

Variables associated with the occurrence of PMI and type 4a MI (as defined by the Fourth UDMI) in the primary cohort study are comprehensively detailed in Table S18. Independent predictors of PMI included creatinine at admission (aOR, 1.20 [95% CI, 1.06–1.35]; *P*=0.003), coronary bifurcation PCI (aOR, 2.62 [95% CI, 1.96–3.51]; *P*<0.001), and total stent length ≥60 mm (aOR, 1.93 [95% CI, 1.39–2.69]; *P*<0.001). Of these variables, only coronary bifurcation PCI (aOR, 3.71 [95% CI, 2.70–5.10]; *P*<0.001) and total stent length ≥60 mm (aOR, 1.87 [95% CI, 1.30–2.68]; *P*<0.001) remained independent predictors of type 4a MI, along with age (aOR, 1.02 [95% CI, 1.01–1.04]; *P*=0.006).

Finally, the procedural risks shown to be independent predictors of PMI defined according to the Fourth UDMI were also independently associated with PMI redefined as a post-PCI ΔcTnI >40% together with an absolute postprocedural value of ≥5 times the 99th percentile URL (Table S19).

## Discussion

The main findings of this first study investigating the incidence and prognostic impact of PMI with and without type 4a MI after NSTEMI are as follows. (1) A significant proportion of NSTEMI patients experienced PMI, as this complication was observed in approximately 4 out of 10 subjects, with no differences between the conventional cTnI and hs-cTnI test periods. (2) PMI was associated with an increased risk of 1-year all-cause mortality and MACEs. (3) Type 4a MI had a significantly increased risk of 1-year all-cause mortality and MACEs, even compared with PMI without type 4a MI criteria. (4) A post-PCI ΔcTnI >20% but ≤40% was associated with 1-year outcomes similar to those observed in patients with post-PCI ΔcTnI ≤20%. Conversely, a post-PCI ΔcTnI increase of >40%, combined with an absolute postprocedural value of ≥5 times the 99th percentile URL, was identified as the best threshold for diagnosing a prognostically relevant PMI. (5) Patients exceeding this threshold had a significant 4-fold and 3-fold increased risk of 1-year all-cause mortality and MACEs, respectively. (6) All of these findings were further validated in an internal cohort and in the sensitivity analysis performed on patients undergoing hs-cTnI measurements.

### Incidence of Periprocedural Ischemic Events in NSTEMI

Our results showed that patients with NSTEMI are frequently susceptible to PMI with and without type 4a MI during the periprocedural period, underscoring the need for a more tailored management approach. Patients with NSTEMI have a remarkably higher incidence of type 4a MI than patients with chronic coronary syndromes, suggesting that specific factors related to the acute setting and subsequent revascularization procedure contribute to the increased risk.^9,14^ In fact, the acute phase of NSTEMI is associated with active inflammatory response, plaque instability, and endothelial dysfunction, all of which favor the development of a prothrombotic state.^9,15^ During PCI, disruption of a vulnerable plaque and subsequent mechanical injury to the coronary endothelium may trigger angiographically evident thrombus formation, as observed in our population, leading to type 4a MI.^9^ A previous study by Lee et al^16^ revealed that patients with NSTEMI who developed post-PCI cTn elevations exhibited coronary lesions with greater lipid length and a higher prevalence of thin-cap fibroatheroma analyzed with optical coherence tomography. During PCI, these high-risk lesions were more frequently associated with distal embolization, contributing to microvascular obstruction and myocardial injury. In addition, the acute inflammatory environment in patients with NSTEMI characterized by the release of inflammatory mediators such as cytokines and chemokines may contribute to an increased susceptibility to myocardial injury by promoting endothelial dysfunction, increasing microvascular resistance, and impairing myocardial perfusion, increasing the risk of no reflow during PCI.^17^ Overall, these factors might contribute to myocardial injury, even in the absence of overt procedural complications (eg, no reflow, coronary dissection, occlusion of a side branch).

Furthermore, the frequent anatomical complexity of coronary lesions in patients with NSTEMI (eg, a higher prevalence of multivessel or left main disease, calcific lesions requiring plaque modification techniques, bifurcations, diffuse disease) might contribute to a higher risk of procedural complications.^18^ Indeed, the high burden and severity of atherosclerotic disease might make PCI technically challenging and increase the likelihood of distal embolization, coronary dissection, or side-branch occlusion, potentially resulting in PMI with or without type 4a MI.^9^

Finally, patients with NSTEMI are typically characterized by a complex clinical profile with multiple comorbidities (age, diabetes, hypertension, chronic kidney disease, etc) and may therefore be more susceptible to periprocedural ischemic events after PCI.^19^

### Prognostic Impact of Type 4a MI

In the present study, patients with NSTEMI experiencing PMI with type 4a MI had a significantly increased risk of adverse clinical outcomes, including mortality at the 1-year follow-up, also compared with patients with PMI without type 4a MI criteria. Type 4a MI represents a critical myocardial insult, triggering an enhanced inflammatory response and potentially exacerbating underlying disease processes.^12^ These factors might ultimately lead to progressive myocardial dysfunction, heart failure, and an increased risk of mortality over time.^20^

The key feature of type 4a MI is the coexistence of elements indicating the onset/exacerbation of myocardial ischemia related to the PCI procedure.^12^ Indeed, our data confirm that the detection of new ischemic changes on ECG or echocardiography and angiographic evidence of the development of a flow-limiting complication provides additional prognostic information compared with the increase in post-PCI cTn levels alone.

The strong prognostic impact of type 4a MI after NSTEMI underscores the need for tailored effective risk stratification and targeted interventions to improve outcomes in this specific patient population. However, it remains uncertain whether mortality and MACEs after type 4a MI in patients with NSTEMI are a consequence of the complexity of the procedure, the vulnerability of the patients, or the extent of iatrogenic cardiac injury.

### Optimizing PMI Diagnosis

In our study, a post-PCI ΔcTnI increase of >40% was identified as the optimal threshold for diagnosing prognostically relevant PMI. Notably, redefining PMI using this threshold, even when combined with an absolute postprocedural cTnI value ≥5 times the 99th percentile URL, was associated with a 4-fold increase in the risk of 1-year all-cause mortality, regardless of the presence of new myocardial ischemia (electrocardiographic, imaging, and angiographic criteria). In contrast, a post-PCI ΔcTnI between 20% and 40% was associated with the same 1-year all-cause mortality and MACE risk as in patients without PMI (post-PCI ΔcTnI ≤20%). This finding could be attributed to a higher rate of false-positive identifications, including patients with mild cTn elevations but without clinically significant myocardial injury. Consequently, including patients with minor cTn elevations, up to a post-PCI change of 40%, in the PMI category could attenuate the association between myocardial injury and adverse outcomes, ultimately reducing its prognostic significance. Furthermore, patients with NSTEMI often have comorbidities such as chronic kidney disease, heart failure, or advanced age, which could account for increases in post-PCI cTn values without necessarily indicating new myocardial injury.^1^ However, this variability did not affect outcomes; the HRs for adverse events at 1 year were adjusted for the major known predictors of outcome in patients with NSTEMI.

The higher threshold identified in our study compared with that proposed by the Fourth UDMI aligns with recent evidence in the context of chronic coronary syndromes.^10^ As a result, the current cTn cutoffs for the diagnosis, which are based on expert consensus rather than robust scientific evidence, may be overly sensitive and might warrant revision in the light of currently available data. This threshold likely represents a critical point of myocardial damage after PCI that cannot be justified solely by comorbidities or reduced cTn clearance. A substantial rise in cTn levels after PCI, even in the absence of clear evidence of new myocardial ischemia, carries considerable prognostic weight. Nonetheless, so far it is still unclear whether management changes might be necessary and which kind of changes might be necessary after the proper identification of these events. In this regard, it could be hypothesized that establishing a ΔcTn threshold associated with adverse outcomes could aid in identifying patients who would benefit the most from changes in management/treatment strategy (eg, prolonged monitoring, intensification of secondary prevention therapy). Furthermore, a post-PCI ΔcTnI >40% for diagnosing PMI could be crucial in identifying patients who may require imaging to confirm or rule out a diagnosis of type 4a MI. Further studies with an appropriate randomized design are needed to address these questions.

Last, the proposed definition of prognostically relevant PMI, a >40% increase 3 to 6 hours after PCI, along with an absolute postprocedural value of ≥5 times the 99th percentile URL, could serve as a valuable clinical end point in future studies exploring management strategies and outcomes in patients with NSTEMI.

### Study Limitations

Our findings should be interpreted considering some limitations. First, 1-year follow-up may be considered relatively short to assess clinical outcomes; a longer follow-up might provide further insights into long-term outcomes and confirm the observed associations. Moreover, 2 different cTnI assays (conventional cTnI and hs-cTnI) were used during the study period. However, because each patient was consistently assessed with the same assay over time, the analysis of ΔcTnI variation does not appear to be influenced by the type of cTnI assay used. Indeed, in our study, the post-PCI ΔcTnI values of patients analyzed with conventional cTnI are similar to those of patients analyzed with hs-cTnI. Furthermore, there was no change in the frequency of PMI with or without type 4a MI between the period of conventional cTnI assay and hs-cTnI assay (Tables S3 and S8). Finally, our results were validated only in an additional cohort of patients from the same sites; external validation is needed to confirm our findings.

### Conclusions

PMI with and PMI without type 4a MI, as defined by the Fourth UDMI criteria, are 2 common PCI-related complications in patients with NSTEMI, with type 4a MI being associated with worse outcomes. We identified a post-PCI change in troponin threshold >40%, combined with an absolute postprocedural value of ≥5 times the 99th percentile URL, as the optimal criterion for diagnosing prognostically relevant PMI. This threshold was strongly linked to increased mortality and MACE, independent of the presence of new myocardial ischemia. These findings may improve risk stratification, guide more tailored management strategies, and ultimately enhance outcomes for patients with NSTEMI undergoing PCI.

## Article Information

### Sources of Funding

None.

### Disclosures

None.

### Supplemental Material

Supplemental Methods

Tables S1–S19

Figures S1–S7

References 21–29
